# Divergent *Nod*-Containing *Bradyrhizobium* sp. DOA9 with a Megaplasmid and its Host Range

**DOI:** 10.1264/jsme2.ME14065

**Published:** 2014-10-04

**Authors:** Kamonluck Teamtisong, Pongpan Songwattana, Rujirek Noisangiam, Pongdet Piromyou, Nantakorn Boonkerd, Panlada Tittabutr, Kiwamu Minamisawa, Achara Nantagij, Shin Okazaki, Mikiko Abe, Toshiki Uchiumi, Neung Teaumroong

**Affiliations:** 1 Center for Scientific and Technological Equipment, Suranaree University of Technology, Muang, Nakhon Ratchasima, Thailand; 2 School of Biotechnology, Suranaree University of Technology, Muang, Nakhon Ratchasima, Thailand; 3 Graduate School of Life Science, Tohoku University, Sendai, Japan; 4 Soil Microbiology Group, Division of Soil Science, Department of Agriculture, Bangkok, Thailand; 5 Graduate School of Agriculture, Tokyo University of Agriculture and Technology, Tokyo, Japan; 6 Graduate School of Science and Engineering, Kagoshima University, Kagoshima, Japan

**Keywords:** *Bradyrhizobium*, *Aeschynomene americana*, broad host range, megaplasmid

## Abstract

*Bradyrhizobium* sp. DOA9, a non-photosynthetic bacterial strain originally isolated from the root nodules of the legume *Aeschynomene americana*, is a divergent *nod*-containing strain. It exhibits a broad host range, being able to colonize and efficiently nodulate the roots of most plants from the Dalbergioid, Millettioid, and Robinioid tribes (7 species of Papilionoideae). In all cases, nodulation was determinate. The morphology and size of DOA9 bacteroids isolated from the nodules of various species of Papilionoideae were indistinguishable from the free-living form. However, they were spherical in *Arachis hypogaea* nodules. GusA-tagged DOA9 also colonized rice roots as endophytes. Since broad-host-range legume symbionts often carry multiple replicons in their genome, we analyzed the replicons for symbiosis genes by electrophoresis. DOA9 carried two replicons, a chromosome (cDOA9) and single megaplasmid (pDOA9) larger than 352 kb. The genes for nodulation (*nodA*, *B*, *C*) and nitrogen fixation (*nifH*) were localized on the megaplasmid. Southern blot hybridization revealed two copies of *nodA* on the megaplasmid, single copies of *nodB* and *C* on the megaplasmid, and one copy each of *nifH* on the chromosome and megaplasmid. These results suggested that *Bradyrhizobium* sp. DOA9 may have the unusual combination of a broad host range, bacteroid differentiation, and symbiosis-mediating replicons.

The genus *Aeschynomene* belongs to the Dalbergioid clade of the subfamily Papilionoideae of the family Fabaceae (19). *Aeschynomene* species establish a symbiotic relationship with bacteria of the genus *Bradyrhizobium* (19, 27). They have been classified into one of three cross-inoculation (CI) groups: CI group 1 species, such as *A. americana* and *A. elaphroxylon*, are only nodulated on their roots by non-photosynthetic bradyrhizobia. CI group 2 species, such as *A. afraspera* and *A. nilotica*, are nodulated on their roots and stems by both non-photosynthetic and photosynthetic bradyrhizobia. CI group 3 species, such as *A. indica* and *A. sensitiva*, are nodulated on their roots and stems by photosynthetic bradyrhizobia. CI group 3 species are nodulated by strains BTAi1 and ORS278 in a Nod factor–independent manner (27). However, *A. indica* (CI group 3) was recently found to be nodulated by a non-photosynthetic strain isolated from *A. americana* (CI group 1). This strain could nodulate several CI groups of *Aeschynomene*, as well as the peanut and mung bean (29).

*Bradyrhizobium* sp. DOA9 (Alphaproteobacteria) was isolated from the root nodules of *A. americana* in Thailand. This strain was assigned to *B. yuanmingense* based on its phenotypic characteristics and multilocus sequences analysis of the 16S rRNA gene and housekeeping genes (*dnaK*, *recA*, and *glnB*). DOA9 was called a divergent *nod*-containing strain based on Southern blot hybridization with the *nod* gene (*nodA*, *B*, and *C*) probes. In addition, on the basis of *nifH* sequence similarities, DOA9 was placed in a cluster of non-photosynthetic bradyrhizobial strains that are able to fix nitrogen in the free-living form. Moreover, it can nodulate a wide range of legumes, including *Macroptilium atropurpureum*, *Arachis hypogaea*, *Vigna radiata*, and *Aeschynomene afraspera* (29). Since information regarding this strain is limited, we investigated the root colonization, infection, and nodulation efficiency of DOA9 in several species from the legume subfamilies Papilionoideae and Mimosoideae, as well as rice. We also examined bacteroids and nodule development. Previous studies demonstrated that symbionts with a broad host range generally carried multiple replicons in their genome (12, 36, 42); therefore, we examined the replicon structure, localization, and copy number of symbiosis genes.

## Materials and Methods

### Bacterial strains and growth conditions

*Bradyrhizobium* sp. DOA9, *B. diazoefficiens* USDA110, and *Mesorhizobium loti* MAFF303099 were cultured at 28°C in HM medium supplemented with l-arabinose (8). GUS-tagged DOA9 was cultured in HM supplemented with streptomycin (200 μg mL^−1^) (29).

### Plant growth and inoculation

Peanut (*Arachis hypogaea*), mung bean (*Vigna radiata*), soybean (*Glycine max* cv. SJ5), siratro (*Macroptilium atropurpureum*), *Sesbania rostrata*, lupin (*Lupinus polyphyllus*), *Desmodium* sp., alfalfa (*Medicago sativa*), *Medicago truncatula*, and *Acacia mangium* seeds were sterilized as described previously (37). *Aeschynomene americana* (a local Thai variety), *A. afraspera*, *A. evenia* (provided by Eric Giraud), and *A. indica* (ecotype Tottori, Japan; ecotype Tomeshi, Japan; and a local Thai variety) were sterilized by incubating in concentrated sulfuric acid for 30 min. The seeds of *Indigofera tinctoria*, *Lotus japonicus*, *Stylosanthes hamata*, *Crotalaria juncea*, *Lespedeza* sp., *Leucaena leucocephala*, *Mimosa pudica*, *Neptunia natans*, and *Samanea saman* were sterilized by incubating in concentrated sulfuric acid for 10 min. The seeds of rice (*Oryza sativa* ssp. *indica*) were sterilized as described previously (26). All seeds were washed with sterilized water and then soaked in sterilized water overnight at ambient temperature. All seeds were germinated on sterilized 0.8% (w/v) water agar for 1 to 2 d at 28°C in the dark. The germinated seeds were transferred onto Hoagland’s agar (15). *Bradyrhizobium* sp. DOA9 was washed with 0.85% NaCl, and optical density at 600 nm (OD_600_) was adjusted to 1 with sterilized water, corresponding to approximately 10^9^ cells mL^−1^. Each seedling was then inoculated with 100 μL of the bacterial culture. All plants were grown at 25°C under a 16 h light/8 h dark cycle at a light intensity of 639 μE m^−2^ s^−1^ for 7 to 14 d (43). These samples were used for microscopic observations. The symbiotic abilities of DOA9 were determined in Leonard’s jars containing sterilized vermiculite and inoculated with 1 mL of bacterial culture, as described above. N-free Hoagland’s solution was added to each jar as required. Plants were harvested after 35 d, used in an analysis of nitrogenase activity by the Acetylene Reduction Assay, and the number of nodules was scored. The dry weights of plants were determined after drying at 70°C for 72 h.

### Microscopic observation of root colonization and nodulation

Root colonization and nodulation were revealed by GUS staining. Samples inoculated with GUS-tagged DOA9 were immersed in GUS assay solution (40 mL 20 mg mL^−1^ X-Gluc in *N,N*-dimethyl-formamide, 20 mg SDS, 2 mL methanol, 0.2 mL 1 M sodium phosphate buffer, and 7.76 mL distilled water) in a vacuum for 120 min and left in this solution for 12 h at 28°C. GUS staining was observed under a light microscope.

### Differential interference contrast and fluorescence microscope observations of isolated bacteroids

Nodules were mashed in bacteroid extraction buffer (125 mM KCl, 50 mM Sodium succinate, 50 mM TES buffer, pH 7.0, with 1% (w/v) BSA) (24). To remove plant cell debris, the suspension was centrifuged at 100×*g* at 4°C for 10 min. To precipitate the bacteroids, the supernatant was centrifuged at 3,000×*g* at 4°C for 10 min. The precipitate was observed using Differential Interference Contrast (DIC) microscopy. Regarding fluorescence microscopy, the bacteroid fraction was first stained with 4′,6-diamidino-2-phenylindole (DAPI; 50 μg mL^−1^) and then with propidium iodide (PI; 2 μg mL^−1^).

### DNA extraction and megaplasmid detection

*Bradyrhizobium* sp. DOA9 total DNA was prepared as described previously (23). Megaplasmids were isolated by electrophoresis (2, 40) with the modifications described here. Bacteria were cultured on HM broth medium (37) with 0.05% (w/v) l-arabinose, 0.05% (w/v) yeast extract for *B. diazoefficiens* USDA110 and *M. loti* MAFF303099, and no l-arabinose for *Bradyrhizobium* strain DOA9, to reduce the production of polysaccharides. USDA110 and DOA9 were cultured at 30°C for 3 to 5 d on a rotary shaker at 200 rpm until the late-log phase was reached. A total of 1% (v/v) of these pre-cultures was then inoculated into new tubes containing HM broth medium. The cultures were incubated for 3 d until the exponential growth phase (the mid-log phase) was reached. MAFF303099 was incubated for 2 d, inoculated into a fresh tube containing HM medium, and then cultured under the same conditions for 24–36 h. Cell pellets were harvested by centrifugation at 3,000×*g* for 10 min. The cells were resuspended in 0.85% (w/v) NaCl to OD_600_=1. They were harvested from 1 mL of a cell suspension and washed with M9 salts (34) containing 0.5 M NaCl, and then with 1 mL 0.1% (w/v) Sarcosyl. The supernatant was removed immediately and the sediment was resuspended in 50 μL of lysis buffer (1 mg mL^−1^ lysozyme, 1 mg mL^−1^ RNase A [33], 0.1% (w/v) bromophenol blue in Trisborate buffer (pH 8.2, 89 mM Tris base, 12.5 mM disodium EDTA, and 8.9 mM boric acid [10], and 40% [v/v] glycerol).

Before sample loading, electrophoresis was performed on a 0.7% agarose gel, which leveled off with 0.5×TBE buffer (4°C) until it touched the gel. The wells were then filled with 50 μL Sodium Dodecyl Sulfate (SDS; 10% [w/v]) mixed with xylene cyanol (1 mg mL^−1^). The current was run for 10–15 min at 100 V with reversed polarity until SDS was 1 cm above the wells. After that, 50 μL of sample mixed with lysis buffer was directly loaded and left for 15 min before 15 μL of Proteinase K (5 mg mL^−1^) in 40% (w/v) glycerol was overlaid. The wells were sealed with melted agarose gel, and 0.5×TBE buffer was then added to cover the gel. After 1 h, electrophoresis was carried out in a cold chamber at 4°C. The current was run at 10 mA for 14 h and at 50 mA for a further 10 h. The DNA in the gel was stained for 30 min in ethidium bromide (0.5 μg mL^−1^) and washed with distilled water before being viewed under UV light.

### Southern blot hybridization

The megaplasmid and chromosomal DNAs separated on the gel were used for the Southern blot hybridization of nodulation (*nod*) and nitrogen fixation (*nifH*) genes as described (29). Briefly, probes for *nodA* (550 bp), *nodB* (530 bp), and *nodC* (1 kb) were obtained through PCR amplification using the genomic DNA of *B. yuanmingense* SUTN6-2, *B. canariense* SUTN7-2, and *B. diazoefficiens* USDA110, respectively. DNA fragments of the respective strains were amplified with the primer pairs nodAYF46/nodAYR595 (*nodA*), nodB26/nodB625 (*nodB*), and nodC195/nodCI (*nodC*) (29). The probe for *nifH* was derived from *B. yuanmingense* using nifHF/nifHI primer pairs (18). DNA probes were labeled overnight at 37°C by random priming, and hybridized with the Digoxigenin (DIG) High Prime DNA Labeling and Detection Starter Kit I (Roche, Switzerland). DNA was capillary-transferred to a Hybond-N^+^ nylon membrane (Amersham, Cardiff, UK) as described previously (34). Low-stringency conditions were used for hybridization: membranes were hybridized at 40°C (*nodA* and *nodC* genes) or 42°C (*nodB* and *nifH*) for 18 h and then washed twice in 2× SSC + 0.1% (w/v) SDS at 25°C for 15 min and in 0.5× SSC + 0.1% (w/v) SDS at 62°C for 15 min.

DOA9 genomic DNA was digested with *Eco*RI, *Eco*RV, *Hind*III, or *Not*I to evaluate the copy numbers of *nodB*, *nodC*, and *nifH*; with *Eco*RI, *Hind*III, *Bgl*II, or *Not*I for *nodA*. Fragments were separated on 1% (w/v) agarose gels before hybridization as described above.

## Results and Discussion

### Nodulation of the subfamily Papilionoideae

**Fig. 1:**
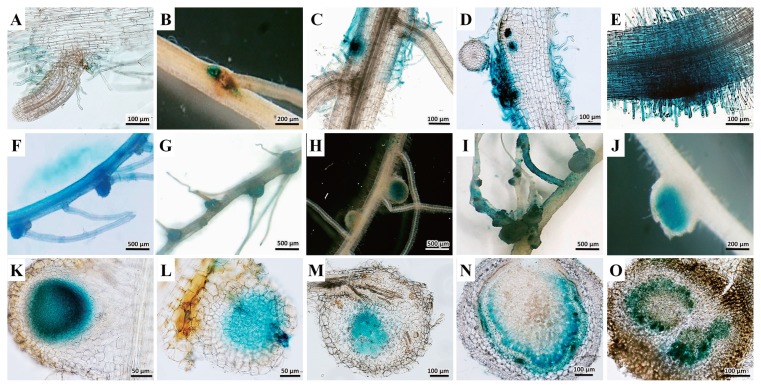
Examples of (A–E) root colonization, (F–J) nodule morphology, and (K–O) thin sections of nodules inoculated with GUS-tagged DOA9 in various legumes. (A, F, and K); *Aeschynomene americana*, (B, G, and L); *Stylosanthes hamate*, (C, H, and M); *Indigofera tinctoria*, (D, I, and N) *Macroptilium atropurpureum*, and (E, J, and O) *Lotus japonicus*.

We tested 19 species and 3 varieties of the ecotypes of *A. indica* (Table 1). DOA9 was able to nodulate the roots of 15 species. Seven of the tested species were effective in terms of nitrogen-fixing efficiency, as indicated by a significant increase in the plant dry weight when inoculated with DOA9 (39) (Table 1 and Table S1 in the supplemental material). An ineffective symbiotic was detected in DOA9 with *S. hamata*, *M. atropurpureum*, *Lepedeza* sp., *L. japonicus*, *L. leucocephala*, and *S. saman*. This strain colonized the lateral root (Fig. 1A–D) and also the root surface (Fig. 1E). In addition, nodulation was related to the colonization sites (Fig. 1F–I). All nodules were determinate (Fig. 1F–J). A thin section of nodules in this group showed achynomenoid types (Fig. 1K–O) (38). DOA9 nodulated both genistoids, but only induced effective nodules on *C. juncea*. Among the Dalbergioids, DOA9 induced effective nodules on *A. americana*, *A. afraspera*, and *A. hypogaea*. Nodules in this group were clearly of the aeschynomenoid type (Fig. 1K–L), which were formed via the crack infection pathway (Fig. 1F and G) (38). This route of infection bypassed some of the complex processes involved in infection via root hairs, which depend on the Nod-factor structure (14). DOA9 induced nodules on all the Millettioids tested, except for *G. max*, even though soybean-nodulating bacteria were distributed among three genera (6, 7). Rhizobia so far known to nodulate Millettioids are all classical rhizobia, mainly slow-growing (“brady-”) types (38). Of the two Robinioids, only *L. japonicus* was nodulated; however, these nodules were ineffective (see Fig. S1 and Table S1). This strain could nodulate the model legume *L. japonicus*. *L. japonicus* may have permitted intercellular infection of the cortex (22). However, DOA9 could not nodulate *S. rostrata*, even though *Sesbania* accepted infection via the crack entry mode (14). Among the Inverted Repeat–Lacking Clade (IRLC), only *M. sativa* was nodulated; however, these nodules were ineffective. These Papilionoideae tribes are generally nodulated with fast-growing α-rhizobia (*Alphaproteobacteria*) and typically show a high degree of specificity between symbiotic partners (38).

### Nodulation of the subfamily Mimosoideae

We tested 4 species (Table 1). Although DOA9 nodulated *L. leucocephala* and *S. saman*, these nodules were not effective. This limited result may have been due to host range specificity, as species of the tribe Mimoseae may be nodulated by β-rhizobia (*Betaproteobacteria*) (3, 11).

*Bradyrhizobium* sp. DOA9 is able to nodulate several species of legumes (Table 1) (29). In addition, *A. americana* plants are commonly nodulated by *Bradyrhizobium* spp. of the cowpea miscellaneous group (41). These findings high-lighted the broad host range characteristics of these bacteria. DOA9 also nodulated many of the Dalbergioids, Millettioids, and Robinioids. DOA9 has been shown to contain divergent *nod*-genes (29), which may facilitate the broad host range nodulation ability. Moreover, the ability of DOA9 to invade roots via the cracks through which lateral roots emerge (29) may allow it to infect a wide variety of legumes; however, we found no infection threads in any sample. These results indicated that DOA9 showed a broad host range.

### Root colonization and infection of rice

**Fig. 2:**
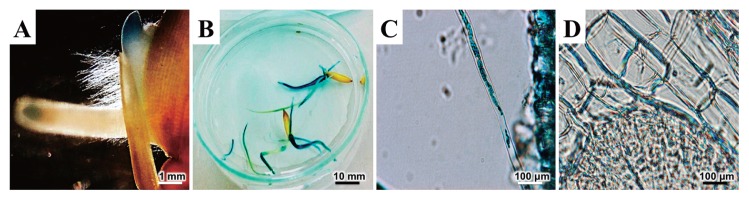
Light microscopic observations of *Oryza sativa* ssp. *indica* seedlings inoculated with GUS-tagged *Bradyrhizobium* sp. DOA9. (A) 1 d after inoculation. (B) 5 d after inoculation. (C) Close-up view of root hair and (D) close-up view of root tissue 5 days after inoculation.

DOA9 colonized the roots and infected the tissues of rice. The expression of GUS was indicated by blue staining (Fig. 2). At 1 d after inoculation, the histochemical staining of β-galactosidase activity revealed the strong colonization of the root cap (Fig. 2A). At 5 d, this strain colonized the entire root (Fig. 2B) and intercellular cells (Fig. 2C and D), as shown in blue. However, we did not measure ARA in rice and its efficiency could not be decided based on plant growth. This should be further analyzed using the Kjeldahl method for the total nitrogen content and compared between inoculated and un-inoculated rice. Previous studies reported that photosynthetic *Bradyrhizobium* strains induced N_2_-fixing nodules on the stems and roots of the genus *Aeschynomene* (28), and also formed a natural endophytic association with the wild rice species *Oryza breviligulata* (5), which grows in association with several aquatic legumes. However, no studies have yet been published on non-photosynthetic bradyrhizobial strains in endophytic association with rice. Therefore, it was interesting that the non-photosynthetic DOA9 could colonize and infect rice. In Thailand, the semi-aquatic *A. americana* frequently grows in association with rice. The genes in DOA9 that are involved in rice infection, which may also play a role in the early interaction between *A. americana* and DOA9, should be examined in more detail. The findings of such a study may reveal a common determinant in the symbiotic interaction between rhizobia and ancestor plants before the symbiotic relationship evolved.

### Microscopic observation of isolated bacteroids

**Fig. 3:**
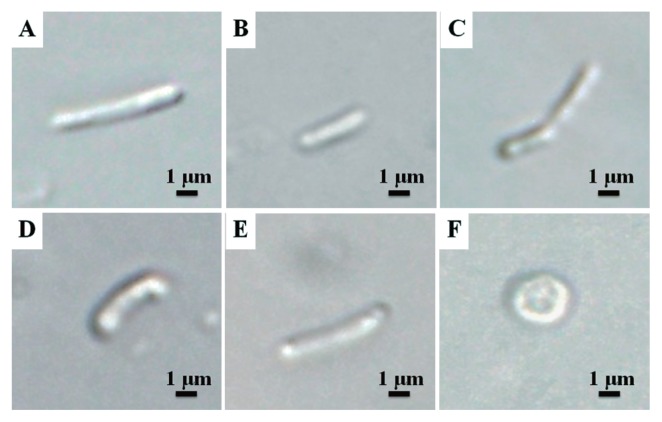
Examples of Differential Interference Contrast (DIC) views of *Bradyrhizobium* sp. DOA9 bacteroids. (A) Free-living. (B–F) Bacteroids isolated from (B) *Indigofera tinctoria*, (C) *Macroptilium atropurpureum*, (D) *Aeschynomene americana*, (E) *Aeschynomene afraspera*, and (F) *Arachis hypogaea*.

**Fig. 4:**
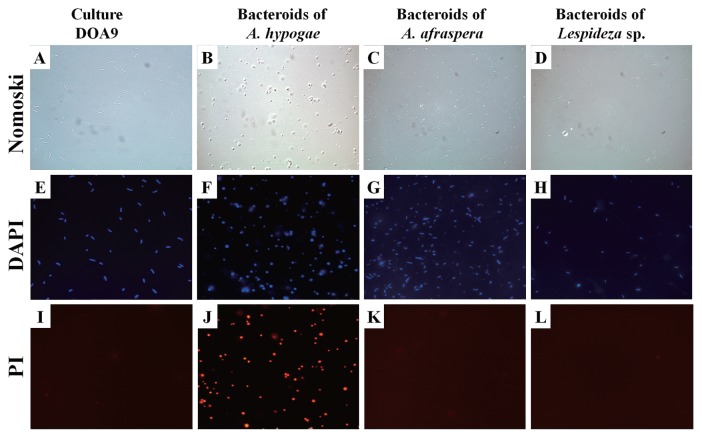
Examples of shapes of (left-hand column) free-living DOA9 bacteria and (other columns) resident bacteroids isolated from (B, F, J) *Arachis hypogaea*, (C, G, K) *Aeschynomene afraspera*, and (D, H, L) *Lespedeza* sp. nodules. DIC, differential interference contrast microscopy; DAPI, fluorescence microscopy with 4′,6-diamidino-2-phenylindole stain; PI, fluorescence microscopy with propidium iodide stain.

Bacteroids isolated from the various species of Papilionoideae were indistinguishable from the free-living form (Figs. 3, and 4). No significant differences were observed in the shape or size of bacteroids between this group and the free-living form (Fig. 3A–E). However, bacteroids from *A. hypogaea* were spherical (Figs. 3F, 4B, 4F, 4J). It is widely accepted that the size and shape of N_2_-fixing bacteroids vary widely and are controlled by the legume host rather than by the rhizobial genotype (31). The bacteroids isolated from the nodules of four out of five Papilionoideae species were not swollen and were the same size (2–4 μm) as the free-living form (Fig. 3A–E).

Furthermore, PI only stained the bacteroids from *A. hypogaea* (Fig. 4J). In addition, no colony appeared when the bacteroid fraction prepared from the nodules of *A. hypogaea* was plated on agar media. Although further quantitative analyses are needed, this result suggests that bacteroids in the nodules of *A. hypogaea* lost their productivity. These characteristics, the absence of PI staining, and the ability to form colonies on agar plates place DOA9 in the group that continues to reproduce after leaving the nodules (25). However, the bacteroids isolated from *A. hypogaea* were spherical and non-reproductive after leaving the nodules. Since the morphology of bacteroids (which is determined by the host legume) has been linked to reproductive viability (25), the host species can have implications for rhizobial evolution (32).

### Megaplasmid detection and hybridization

**Fig. 5:**
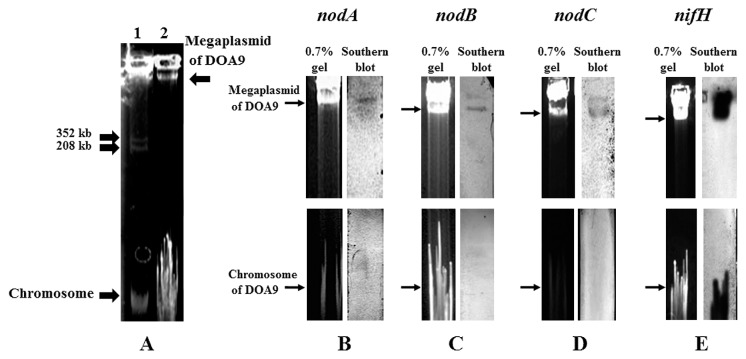
Determination of *Bradyrhizobium* sp. DOA9 replicons and symbiosis genes. (A) Megaplasmid profiles: lane 1, *Mesorhizobium loti* MAFF303099 (208 and 352 kb); lane 2, DOA9; (B–E) Southern blot hybridization signals of nodulation and N-fixation genes on the megaplasmid and chromosome of DOA9 under low stringency conditions: (B) *nodA*; (C) *nodB*; (D) *nodC*; (E) *nifH*.

We successfully extracted both chromosomal and megaplasmid DNAs. The megaplasmid size was estimated from the plasmid profile of *M. loti* MAFF303099, which contained megaplasmids of 208 and 352 kb in size (16) (Fig. 5A lane 1). DOA9 revealed one megaplasmid, which was larger than 352 kb (Fig. 5A lane 2). The megaplasmid hybridized with all the probes for *nodA*, *nodB*, *nodC*, and *nifH* (Fig. 5B–E). The *nifH* probe also hybridized with the chromosomal DNAs (Fig. 5E).

Broad-host-range symbionts generally carry multiple replicons on their genome. *Sinorhizobium* sp. NGR234, which has an extremely broad host range, has three replicons: a symbiosis plasmid, megaplasmid, and chromosome (12); *S. fredii* USDA257 has a chromosome and plasmid (36); *S. fredii* HH103 has one chromosome and five plasmids (42); DOA9 has a chromosome (cDOA9) and megaplasmid (pDOA9) larger than 352 kb. A large number of the natural isolates of *Rhizobium* spp. have been shown to carry various large plasmids (21). Genes for a few functions have been detected on these large plasmids, particularly the *nod* and *nif* genes of fast-growing rhizobia. In contrast, the published genomes of *Bradyrhizobium* spp. are composed of only a chromosome, except for *Bradyrhizobium* sp. BTAi1, which also harbors a plasmid of 228 kb (9). There have not yet been any studies published on symbiosis genes localized on the plasmids of *Bradyrhizobium* (21). The symbiotic islands of *B. diazoefficiens* USDA110 have been located on a chromosome, while the symbiosis genes of *Sinorhizobium* sp. were located on plasmids (13). This characteristic may contribute to the broad host range of these rhizobia. It will be interesting to find out whether symbiosis genes on plasmid have extended their host range.

### Copy number

**Fig. 6:**
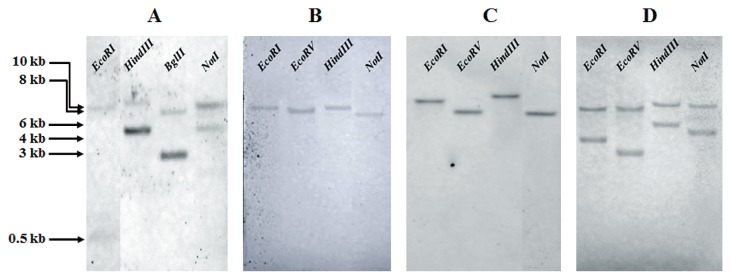
Determination of copy numbers of (A) *nodA*, (B) *nodB*, (C) *nodC*, and (D) *nifH* by Southern blot hybridization. *Bradyrhizobium* sp. DOA9 genomic DNA was digested with the restriction enzymes shown, and the blot was hybridized with probes for *nodA* (from *B. yuanmingense* SUTN6-2), *nodB* (from *B. canariense* SUTN7-2), *nodC* (from *B. japonicum* USDA110), and *nifH* (from *B. yuanmingense* SUTN6-2).

At least two fragments were detected using the probe for *nodA* (Fig. 6A). This result suggested that *nodA* on the megaplasmid was a single or two copies. Regarding *nodB* and *nodC*, a single fragment was detected on each lane, suggesting that these two genes may be a single copy on the megaplasmid (Fig. 6B, and C). At least two fragments were detectable when probed by *nifH*. The result supported the two *nifH* genes being separately located on the megaplasmid and chromosome (Figs. 5E, and 6D).

DOA9 has at least two copies of *nifH*, one on the megaplasmid and one on the chromosome. Two copies of *nifH* were also shown to be present in photosynthetic *Bradyrhizobium* ORS278 and BTAi1 (30), and one copy in *B. diazoefficiens* USDA110 (17), in all cases on the chromosome. We already sequenced *nodA* of DOA9 (Accession No. DF 820426.1), which was the corresponding fragment of the *nodA* probe used in this study, and no restriction sites of *Eco*RI, *Hind*III, *Bgl*II, or *Not*I were identified; therefore, the hybridization data probed by *nodA* suggested that DOA9 may have at least two copies of *nodA* on the megaplasmid. An increase in the copy number of the symbiotic region has been indicated to promote the plant phenotype. The inoculation of alfalfa with a moderate increase in the copy number of the symbiotic region of *S. meliloti* resulted in enhanced plant growth (4).

Therefore, this is the first study on the symbiosis genes of *Bradyrhizobium* sp. located on a megaplasmid. A detailed investigation of both replicons of DOA9 is warranted. Previous studies on the host range of rhizobia have focused on *Sinorhizobium* sp. strain NGR234, *S. fredii* USDA257, and *S. fredii* HH103. Since the genome sequences of these three strains share a high degree of synteny (35, 36, 42), the host range of rhizobia may be related to the number of specialized protein secretion systems they carry (35). Therefore, a future study is needed to determine and compare the protein secretion systems of DOA9.

## Conclusion

Since many of the photosynthetic bradyrhizobial (PB) strains have been reported to be naturally endophytic with rice (5), we concluded that rice may have evolved approximately 120 million years before legumes (1, 20). Thus, PB strains may also be an ancestor of non-photosynthetic bradyrhizobia (41). In the present study, DOA9 exhibited the characteristic of a broad host range in legume and rice infections. Therefore, DOA9 may represent the interval of evolution in the nod factor-independent symbiotic system, but may have later lost photosynthetic activity. Moreover, DOA9 is a divergent *nod*-containing strain rendering the incomplete production of nod factors. This may also support DOA9 associating with host plants in a nod-independent manner since and during its evolution. To more clearly understand the evolution of this group, whole genome assessments, symbiotic genes comparisons and disruptions, as well as protein secretion systems and lipopolysaccharide structures should be explored in more detail.

## Supplementary Information



## Figures and Tables

**Table 1:** Nodulation by *Bradyrhizobium* sp. DOA9 and bacteroid morphology in various legumes

Plants	Nodulation^a^	Nodule type	Bacteroid^c^
**Papilionoideae**			
**Genistoids**			
*Lupinus polyphyllus*	(+)	determinate	unswollen
*Crotalaria juncea*	+	determinate	unswollen
**Dalbergioids**			
*Aeschynomene americana* (a local Thai variety)	+	determinate	unswollen
*Aeschynomene indica* (ecotype Tottori, Japan)	−	−	−
*Aeschynomene indica* (ecotype Tomeshi, Japan)	−	−	−
*Aeschynomene indica* (a local Thai variety)	−	−	−
*Aeschynomene afraspera*	+	determinate	unswollen
*Aeschynomene evenia*	−	−	−
*Arachis hypogaea*	+	determinate	swollen
*Stylosanthes hamata*	(+)	determinate	swollen
**Millettioids**			
*Glycine max* (cv. SJ5)	−	−	−
*Macroptilium atropurpureum*	(+)	determinate	unswollen
*Vigna radiata*	+	determinate	unswollen
*Desmodium* sp.	+	determinate	unswollen
*Lespedeza* sp.	(+)	determinate	unswollen
*Indigofera tinctoria*	+	determinate	unswollen
**Robinioids**			
*Lotus japonicus* (ecotype Miyagi, Japan)	(+)	determinate	unswollen
*Sesbania rostrata*	−	−	−
**IRLC (inverted repeat–lacking clade)**			
*Medicago truncatula*	−	−	−
*Medicago sativa*	(+)	determinate	unswollen
*Trifolium repens* (white clover)	−	−	−
**Mimosoideae**			
**Mimoseae**			
*Mimosa pudica*	−	−	−
*Leucaena leucocephala*	(+)	determinate	unswollen
*Neptunia natans*	−	−	−
**Ingeae**			
*Samanea saman*	(+)	determinate	unswollen

a +, positive with effective nodules; (+), positive with ineffective nodules; −, negative.
