# Supplementary material for: Divergent Nod-Containing Bradyrhizobium sp. DOA9 with a Megaplasmid and its Host Range
Source: Microbes Environ. 2014 Oct 4;29(4):370–6. doi: 10.1264/jsme2.ME14065 (PMC4262360; doi:10.1264/jsme2.ME14065)
Supplement: Supplementary file 1 [file 29_370_s1.pdf]

Figure legends-supplemental data

Fig. S1 GUS-tagged Bradyrhizobium sp. DOA9 induced ineffective nodules on Lotus japonicus. (A, B) Nodules. (C) Thin section of a nodule.

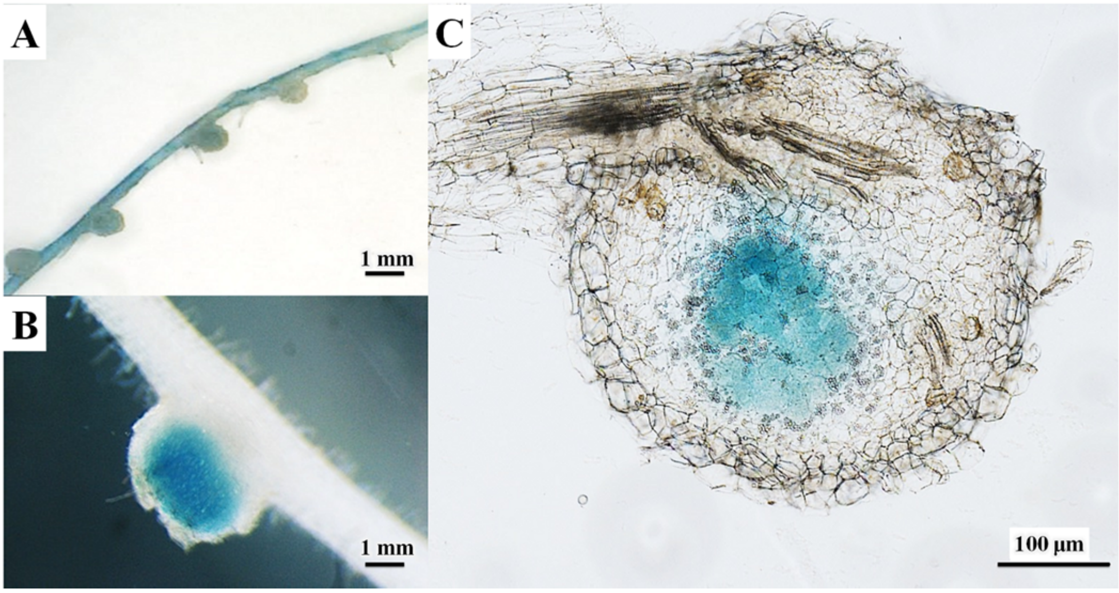

Table S1. Symbiotic phenotype of *Bradyrhizobium* sp. DOA9 with various legumes.

Plants	Strain	No. of root (Nodules/plant)	Plant dry weight (g/plant)	Fixation activity[c] (nmol /g plant DW)
C. juncea	No inoculum	0.00	135.00±1.13	0.00
	DOA9	9.56±7.51	249.66±66.21*	0.03±0.04
A. americana	No inoculum	0.00	5.30±0.32	0.00
	DOA9	7.78±4.60	13.81±2.75**	1,070.53±490.35
A. afraspera	No inoculum	0.00	152.85±16.34	0.00
	DOA9	36.83±5.48	431.33±51.02**	1.50±0.22
A. hypogaea	No inoculum	0.00	933.60±11.96	0.00
	DOA9	67.00±11.52	1,538.18±116.88**	0.91±0.18
S. hamata	No inoculum	0.00	7.03±1.65	0.00
	DOA9	11.00±1.87	6.48±1.16	1,144.17±219.74
M. atropurpureum	No inoculum	0.00	56.93±6.04	0.00
	DOA9	17.33±4.13	46.72±10.96	98.03±16.20
V. radiata	No inoculum	0.00	284.20±2.90	0.00
	DOA9	16.14±1.40	363.80±36.41**	4.81±0.77
Desmodium sp.	No inoculum	0.00	821.50±10.85	0.00
	DOA9	57.00±9.42	1,427.17±105.76**	0.80±0.17
Lepedeza sp.	No inoculum	0.00	13.03±1.20	0.00
	DOA9	5.63±1.94	9.50±2.26	395.67±189.73
I. tinctoria	No inoculum	0.00	11.53±0.95	0.00
	DOA9	9.56±1.35	30.13±4.24**	72.42±20.61
L. japonicus	No inoculum	0.00	7.45±0.54	0.00
	DOA9	2.58±1.63	7.63±0.48	248.17±139.13
L. leucocephala	No inoculum	0.00	152.50±16.12	0.00
	DOA9	1.33±0.58	135.12±13.16	27.40±22.22
S. saman	No inoculum	0.00	355.75±20.00	0.00
	DOA9	67.50±14.55	428.07±52.43	237.85±77.50

The number of nodules, plant dry weight and acetylene reduction assay was measured 35 days after inoculation.

** and * are mean significant difference of plant dry weight in each plant host at 1% and 5%, respectively according to t-test.
